# Sleep‐disordered breathing in heart failure: facts and numbers

**DOI:** 10.1002/ehf2.12193

**Published:** 2017-07-17

**Authors:** Charlotte Pietrock, Stephan von Haehling

**Affiliations:** ^1^ Division of Cardiology and Metabolism: Heart Failure, Cachexia and Sarcopenia, Department of Internal Medicine and Cardiology Charité Medical School Berlin Germany; ^2^ Department of Cardiology and Pneumology University Medical Centre Göttingen Göttingen Germany

**Keywords:** Sleep apnoea, Heart failure, Therapy

## Abstract

Sleep‐disordered breathing has a high prevalence in the general population, but is especially prominent in patients with heart failure (HF). HF and sleep‐disordered breathing share a bidirectional relationship, with sleep‐disordered breathing being both cause and effect of poor cardiac functioning. The high inter‐individual variability of symptom presentation can impede the clinical diagnostic process. Polysomnography is the gold‐standard method of diagnosing sleep‐disordered breathing. Therapy of sleep‐disordered breathing should always consist of optimizing the treatment of the underlying disorder of HF. Additional therapeutic measures include continuous positive airway pressure ventilation therapy. New therapeutic options using neurostimulation are yielding promising results; however, long‐term benefits still need to be confirmed.

## Introduction

Sleep‐disordered breathing is highly prevalent in patients with heart failure (HF) and has a strong impact on clinical outcome. Their relationship is antagonistic: poor cardiac function can induce a breathing disorder, and correspondingly, sleep‐disordered breathing can adversely affect the progression of HF. This reinforces a pathophysiological circulus vitiosus. Due to the high prevalence, high morbidity, and high mortality of HF, knowing, diagnosing, and treating the common comorbidity of sleep‐disordered breathing become indispensable.

Sleep‐disordered breathing includes all disturbances in respiratory behaviour during sleep. The pre‐eminent disorders are obstructive sleep apnoea (OSA), central sleep apnoea (CSA), and mixed sleep apnoea, which presents with components of both OSA and CSA. All three forms are characterized by episodes of hypopnoea and/or apnoea, with disease severity increasing with the number of events.^1^, ^2^, ^3^, ^4^, ^5^ In this context, hypopnoeas are defined as a reduction in nasal flow of ≥50% for ≥10 s combined with arousal or a decline in oxygen saturation ≥4 percentage points. Apnoeas are prolonged pauses in breathing during sleep with a duration of ≥10 s occurring >5 per hour.^3^ They are likewise associated with a drop in oxygen saturation.

### Obstructive sleep apnoea

The most common form of sleep‐disordered breathing, OSA, is primarily caused by an obstruction of the upper respiratory tract that results in repeated interruptions of the normal breathing process during sleep. Patients with OSA are often anatomically predisposed to smaller pharyngeal breathing tracts as a result of obesity, enlarged tonsils, adenoids, or tissue irregularities.^6^, ^7^ During sleep, the pharyngeal dilator muscle activity, which compensatory ensures a regular air passage during wakefulness, is diminished. This leads to a partial or complete airway obstruction, producing hypopnoeas or apnoeas, respectively. The resulting hypoxia and hypercapnia act as strong central stimulants and provoke a reacquisition of normal breathing and/or arousal.^3^, ^4^, ^8^, ^9^, ^10^ Patients are not usually aware of such episodes of arousal, but they still cause stress.

### Central sleep apnoea

The central form of apnoea is caused by a dysfunction of the ventilatory control system. The patency of the respiratory tract is functional, but the ventilatory effort, the essential central trigger for breathing, is intermittently disrupted. This malfunction can originate from a variety of neurological diseases^11^, ^12^, ^13^, ^14^ or HF.^1^, ^3^, ^5^, ^15^, ^16^, ^17^, ^18^, ^19^, ^20^, ^21^, ^22^, ^23^, ^24^ In HF, the accepted hypothesis is that pulmonary congestion, (which is further aggravated in a horizontal sleeping position) due to a higher left ventricular filling pressure, activates lung vagal irritant receptors causing hyperventilation and hypocapnia.^3^, ^5^, ^19^ The paCO_2_ threshold to stimulate the central respiratory centre is not surpassed. In consequence, the respiratory muscles are insufficiently innerved, and normal breathing surceases.^10^ Cheyne–Stokes respiration is a frequent breathing pattern observed in CSA; it is characterized by periodic fluctuations between hypopnoea/apnoea and hyperventilating crescendo and decrescendo respiratory phases.^1^, ^3^, ^16^ However, Cheyne–Stokes respiration is not a prerequisite for a diagnosis of CSA. This respiration pattern can even be observed during wakefulness and exercise in patients with advanced chronic HF.^25^, ^26^ Central apnoeas may also appear concomitantly to obstructive apnoeas, resulting in mixed sleep apnoea.

The pathophysiological consequences of regular hypopnoea and apnoea phases are a permanent increase in sympathetic nervous system activity. An increased heart rate, diminished heart rate variability, higher blood pressure, and increased cardiac oxygen demand ensue.^3^, ^5^, ^24^, ^27^, ^28^ Additionally, oxidative stress may be triggered as a result of the repeated hypoxemia and reoxygenation processes in sleep‐disordered breathing.^29^, ^30^, ^31^, ^32^ The collaborative impact of these factors can contribute to pathological remodelling processes of the myocardium.^1^, ^33^, ^34^, ^35^

## Clinical presentation

Common symptoms of sleep‐disordered breathing are unrestful sleep, fatigue, hypersomnolence, and cognitive dysfunction. Other clinical signs include headaches, nocturia, erectile dysfunction, reduction of libido and witnessed apnoea, or gasping.^1^, ^3^ Rhonchopathy is especially prevalent in OSA patients, and CSA patients can present with cardiac or neurological manifestations of the underlying condition.^1^ There is a high inter‐individual variability in the presentation of symptoms in sleep‐disordered breathing, with disease severity not necessarily corresponding to symptom severity. Some patients do not present with any symptoms at all. This is particularly true in patients with symptomatic HF, in whom 50–70% of patients are affected by sleep‐disordered breathing.^21^, ^36^, ^37^ Whilst patients with less symptomatic status more often present with OSA, CSA is becoming more prevalent in advanced stages of HF, that is, in patients in New York Heart Association classes III and IV.^22^ Even more problematic is the fact that typical signs of sleep‐disordered breathing may be absent in patients with HF or they may overlap with symptoms of HF itself, therefore rendering the differential diagnosis difficult. Even screening tools that work well in subjects without HF such as the Epworth Sleepiness Scale have not proven reliable in patients with HF.^38^ Other screening questionnaires are the Berlin Questionnaire^39^, ^40^ and the STOP‐Bang Sleep Apnea Questionnaire,^41^, ^42^, ^43^, ^44^ but both have not been validated in patients with HF. The severity of the disease can be screened for using two indices: The Apnoe–Hypopnoe Index (AHI) and Oxygen Desaturation Index (ODI). The AHI represents the number of apnoeas and hypopnoeas per hour of sleep; an AHI <5 per hour is considered physiological.^45^ An AHI 5–15 per hour defines a mild, AHI 15–30 per hour a moderate, and AHI >30 per hour a severe form of sleep‐disordered breathing. The ODI reflects the number of respiratory events that result in a reduction in oxygen saturation of ≥4%.

## Clinical diagnosis

The predominant method of diagnosing sleep‐disordered breathing is via polysomnography that is used in the sleep lab or its more elementary counterpart, polygraphy, that can be used on an outpatient basis. A polysomnography is a multi‐parametric sleep study that monitors respiratory airflow, oxygen saturation (via pulse oximetry), thoracic and abdominal respiratory effort, rhonchopathy, heart activity (via electocardiography), skeletal muscle behaviour (via electromyography), electrical brain activity (via electroencephalography), and eye movement (via electro‐oculography) during sleep. A polygraphy only includes the recordings of respiratory airflow, oxygen saturation, and thoracic and abdominal movement. The collected parameters are then analysed to obtain AHI, ODI, and cardiac, breathing, or sleep irregularities. The usual approach is to screen patients for the presence of sleep‐disordered breathing using polygraphy and to refer those with elevated AHI/ODI values for polysomnography.

## Prevalence of sleep‐disordered breathing in heart failure

The prevalence of moderate to severe sleep‐disordered breathing is currently estimated to be 10–17% in the adult male population and 3–9% in the adult female population in the United States.^46^ In patients with HF, the prevalence is significantly higher.^21^, ^22^, ^47^, ^48^ In one study, patients with chronic HF (New York Heart Association ≥II or left‐ventricular ejection fraction ≤40%) 40% were diagnosed with CSA and 35% with OSA.^21^

Central sleep apnoea is a primary comorbidity in HF,^1^, ^3^, ^5^, ^15^, ^16^, ^17^, ^18^, ^20^, ^23^ increasing in prevalence with deteriorating cardiac function.^21^, ^22^, ^48^ Chronic HF is recognized as an independent risk factor for the development of CSA.^1^, ^5^ Concurrently, CSA adversely affects the progression of HF, predominantly through the permanently increased sympathetic nervous system activity and its consequences.^5^ Likewise, OSA is also more prevalent in patients with HF as compared with the general population.^1^, ^24^ It is also its own risk factor for cardiovascular disease.^4^, ^49^, ^50^, ^51^ The presence of OSA fundamentally contributes to myocardial failure and earlier mortality in patients with HF.^4^ Although awareness among physicians of sleep‐disordered breathing is growing, clinically significant sleep‐disordered breathing is still vastly under‐diagnosed and even available tools may be underutilized.^3^, ^52^, ^53^

## Therapy of sleep‐disordered breathing

The core therapeutic measure in treating HF patients with the comorbidity of sleep‐disordered breathing is always ensuring the patient's receiving of optimal HF treatment. If the sleep‐disordered breathing persists, other additional therapeutic measures may be employed.^3^, ^54^

In OSA, continuous positive airway pressure (CPAP) ventilation therapy is recommended.^54^ The continuous air pressure provided by an overnight mask ensures improved ventilation and ameliorates the patient's symptoms. Other options are oxygen supplementation, bi‐level positive airway pressure, or adaptive servo‐ventilation (ASV) therapy, which appropriately adapts the positive airway pressure according to changes in the patient's breathing pattern, thereby enhancing the ventilatory efficacy. Unfortunately, none of these interventions positively impacts the outcome of HF.^55^

Patients with CSA may also benefit from CPAP, bi‐level positive airway pressure, or nocturnal oxygen supplementation therapy.^54^ Several studies have demonstrated a therapeutic effectiveness of chronic resynchronization therapy in patients with HF and CSA.^56^, ^57^, ^58^ A recent study demonstrated that ASV improved CSA, but significantly increased cardiovascular and all‐cause mortality.^24^ Therefore, ASV is no longer recommended for CSA patients with chronic HF.

Modern, more invasive, yet promising interventions to improve sleep‐disordered breathing are nerve stimulations. In patients with OSA, hypoglossal neurostimulation has been shown to reduce AHI and symptom severity by reducing the obstruction of the upper respiratory tract.^59^, ^60^ Further research is necessary, but current results deem it an auspicious therapeutic measure, especially for OSA patients intolerant or non‐responsive to CPAP therapy. Unilateral phrenic nerve stimulation is a further treatment option for patients with CSA. Current results indicate a significant reduction in AHI, symptom improvement, and increased quality of life in patients receiving the implantable device; nonetheless, long‐term effects still need to be established.^61^

## Conflict of interest

SvH has been a paid consultant for Respicardia, Vifor, Roche and BRAHMS. CP does not have a conflict of interest.
